# Development of tumor-specific caffeine-potentiated chemotherapy using a novel drug delivery system with Span 80 nano-vesicles

**DOI:** 10.3892/or.2015.3761

**Published:** 2015-01-29

**Authors:** HIROSHI NAKATA, TATSUHIKO MIYAZAKI, TOMOYUKI IWASAKI, ATSUSHI NAKAMURA, TERUKI KIDANI, KENSHI SAKAYAMA, JUNYA MASUMOTO, HIROMASA MIURA

**Affiliations:** 1 Department of Orthopaedic Surgery, Ehime University Graduate School of Medicine, To-on, Ehime, Japan; 2 Division of Pathology, Gifu University Hospital, Gifu-city, Gifu, Japan; 3 Division of Medical Bioscience, Ehime University Integrated Center for Science, To-on, Ehime, Japan; 4 Division of Orthopaedic Surgery, Minami Matsuyama Hospital, Matsuyama, Ehime, Japan; 5 Department of Analytical Pathology, Ehime University Graduate School of Medicine, To-on, Ehime, Japan

**Keywords:** Span 80 vesicles, drug delivery system, osteosarcoma, LM8, caffeine-potentiated chemotherapy

## Abstract

In recent years, chemotherapy with caffeine has manifested potently high efficacy against osteosarcoma, although adverse effects have been observed. Recently, we developed a novel drug delivery system (DDS) with nonionic vesicles prepared from Span 80 which have promising physicochemical properties as an attractive possible alternative to commonly used liposomes. Herein, we demonstrated that tumor-specific caffeine-potentiated chemotherapy for murine osteosarcoma administered by a novel DDS with Span 80 nano-vesicles showed significant antitumor effects as well as limited adverse effects. The osteosarcoma cell line, LM8, was transplanted into C3H/HeJ mice which then were administered therapeutic agents. Ifosfamide (IFO) was employed as well as caffeine as an enhancer. Span 80 vesicles containing IFO and/or caffeine were freshly prepared. On days 0, 2 and 4, different combinations of the agents were administered to mice: IFO alone (direct i.v.), IFO vesicles (IV), IV + caffeine, IV + caffeine vesicles (CV), PBS alone vesicles (PV), and PBS alone as negative control (PBS i.v.). Then, the mice were sacrificed on day 7. Antitumor effects of the reagents were also analyzed *in vitro*. Moreover, fertility examination was performed. *In vitro*, a combination of IV+CV showed significant induction of apoptosis in the early phase. Tumor volumes in the IV+CV group were significantly reduced compared with the other groups. Histological analyses showed that the IV and IV+CV groups had significantly lower viable tumor areas. The IFO direct i.v. group showed a certain grade of renal injury as well as marked suppression of spermatogenesis, while the IV or IV+CV group showed no marked changes. The fertility test revealed that the male mice with IV+CV administration had normal fertility, and no malformations were detected in their progeny. This DDS model is of potential importance for clinical application in the therapy of metastatic osteosarcoma.

## Introduction

At present, osteosarcoma cases with metastasis have poor prognosis (1,2). In recent years, caffeine-potentiated chemotherapy, which is chemotherapy with caffeine dosage against malignancies, has manifested potently high efficacy (3,4). Nevertheless, this method may induce side effects with individual diversity (5). On the other hand, there have been numerous developments in novel drug delivery systems (DDS) for drug carriers for the treatment of various diseases (6–8).

Recently, we demonstrated that nonionic vesicles prepared from Span 80 have promising physicochemical properties, such as high membrane fluidity associated with low phase transition temperature, which make them an attractive possible alternative to the commonly used liposomes. Span 80 is generally known as sorbitan monooleate, although commercial Span 80 is a heterogeneous mixture of sorbitan mono-, di- tri, and tetra-esters that may contribute to high fluidity and vascular permeability (9–11).

A successful therapeutic model using the DDS with Span 80 vesicles and immobilized polysaccharides in transplanted colon cancer cell lines was reported (12). Herein, we showed that tumor-specific caffeine-potentiated chemotherapy for murine osteosarcoma using a novel DDS with Span 80 nano-vesicles showed significant antitumor effects, as well as limited adverse effects.

## Materials and methods

### Antitumor agents

As therapeutic agents, ifosfamide (IFO) was employed as well as caffeine sodium benzoate (CSB) as an enhancer. IFO was obtained from Shionogi & Co. Ltd. (Osaka, Japan), and CSB was purchased from Maruishi Pharmaceutical Co. Ltd. (Osaka, Japan).

### Preparation of Span 80 nano-vesicles

Span 80 nano-vesicles, which contained IFO and/or caffeine, were freshly prepared as previously described (12). Briefly, materials for assembling nano-vesicles containing Span 80 (sorbitan monooleate) and Tween-80 [polyoxyethylene (20) sorbitan monooleate] were purchased from Wako Pure Chemical Industries Ltd. (Osaka, Japan). Cholesterol, which worked as the stabilizer of the membrane, was obtained from Nacalai Tesque Inc. (Kyoto, Japan), and polyethylene glycol, used as a stealth modifier against macrophages, was acquired from NOF Corp. (Tokyo, Japan). The solvents, normal hexane and normal saline, were obtained from Nacalai Tesque Inc. and Otsuka Pharmaceutical Factory Inc. (Naruto, Japan), respectively. All processes were performed under sterilized conditions.

The two-step emulsification method was employed to make and purify the nano-vesicles. Span 80 (190 mg) and cholesterol (9 mg) were dissolved in 4.5 ml of hexane by homogenization with a micro-homogenizer, NS-310E (Microtec Co. Ltd., Funabashi, Japan), at 15,000 rpm for 30 sec in a sterilized brown glass bottle. Sequentially, 40 mg/ml of IFO and/or 50 mg/ml of CSB, which were dissolved in 4.5 ml of normal saline, were dripped into the bottle of Span 80 material followed by homogenization for 3 min, resulting in production of the first emulsion. As a negative control, phosphate-buffered saline (PBS) was employed instead of the antitumor reagents. The emulsion was evaporated by a rotary vacuum evaporator, N-1000 (Tokyo Rikakikai Co. Ltd., Tokyo, Japan), in a vacuum flask on a water bath at 37°C followed by homogenization with 72 mg of Tween-80 and 25 mg of DSPE-020CN at 3,500 rpm for 5 min, which produced the second-stage emulsion.

The second emulsion was centrifuged using an ultracentrifugation equipment (CP70G with RP65T rotor, Hitachi Koki Co. Ltd., Tokyo, Japan). After aspiration of the supernatant, the pellet consisting of the Span 80 vesicles was weighed and suspended in normal saline at a concentration of 20% w/v, resulting in IFO Span 80 vesicles (IV), CSB Span 80 vesicles (CV), and PBS alone Span 80 vesicles (PV). Immediately prior to administration *in vivo* or *in vitro*, these suspensions were extruded by a custom made extruder (EP Tech Co. Ltd., Ibaraki, Japan), which was equipped with a drain disk of 100-μm thickness and a Nucleopore membrane^®^ of 100-nm pore size (GE Healthcare Japan Co. Ltd., Tokyo, Japan) in order to control the size of the vesicles. The diameter of the vesicles was evaluated by the Dynamic Light Scattering device, DLS-6000EW (Otsuka Electronics Co. Ltd., Osaka, Japan). The average diameter of the vesicles was 117 nm.

### In vitro evaluation of the antitumor effects of the nano-vesicles

The murine osteosarcoma cell line, LM8, was obtained from the Riken BRC Cell Bank (Tsukuba, Ibaraki, Japan). LM8 cells (1 ml) in Dulbecco’s modified Eagle’s medium (1×10^5^ cells/ml suspension) were plated per well and cultured in 24-well culture dishes (Nunc #142475; Thermo Scientific Inc., Waltham, MA, USA) for a few days until the tumor cells were semi-confluent. Then, antitumor agents with or without Span 80 vesicles, consisting of PV, IV, CV, IFO (direct administration), CSB (direct administration), IV+CSB, IFO+CV and IV+CV, were administered at 100 μl/well. Cells were incubated with the antitumor agents at 37°C for 1 or 2 h, and then the cells were harvested and evaluated for apoptosis and cell viability, respectively. Assessments of each condition were examined in triplicate.

### Evaluation of cell viability

Cells were incubated with 0.4% trypan blue stain (Life Technologies Japan Co. Ltd., Tokyo, Japan) in PBS for 1 min, and then images of each well were captured by an inverted microscope, TMS (Nikon Instruments Co. Ltd., Tokyo, Japan), and a DP-25 digital camera (Olympus Co. Ltd., Tokyo, Japan). Non-viable cells in each image were evaluated by Image Pro Plus^®^ software (Media Cybernetics Inc., Rockville, MD, USA) in triplicate.

### Apoptosis detection by propidium iodide (PI) method

Briefly, cells were suspended in 500 μl of ice-cold Hank’s Balanced Saline Solution (HBSS; Sigma-Aldrich Japan, Tokyo, Japan), followed by fixation with 4.5 ml of 70% EtOH at −20°C (13). Fixed cells were centrifuged at 400 × g for 10 min, pellets were re-suspended in extraction buffer pH 7.8 which contained 45 mM Na_2_HPO_4_ (Nacalai Tesque Inc.), 25 mM citric acid (Wako Pure Chemical), and 0.1% Triton X-100 (Wako Pure Chemical) at 37°C for 25 min. Then, 300 μl of staining solution pH 6.8 containing 10 mM PIPES (Sigma-Aldrich Japan), 100 mM NaCl (Nacalai Tesque Inc.), 2 mM Mg_2_Cl (Wako Pure Chemical), 0.2% Triton X-100, 50 mg/ml PI (Sigma-Aldrich Japan), and 50 U/ml RNAse H (0.6 mg/ml, Takara Bio Co., Ltd., Otsu, Japan) were added to the cell suspension, and the fluorescence intensity was evaluated and analyzed in triplicate by the FACStation^®^ and CellQuest^®^ software (Becton-Dickinson, Franklin Lakes, NJ, USA).

### Murine osteosarcoma therapeutic model

**Figure 1:**
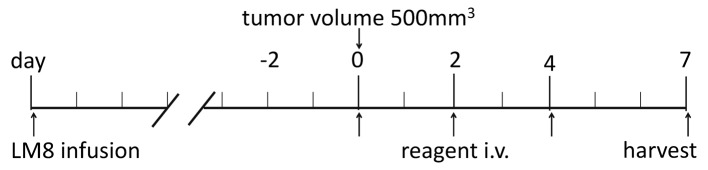
The *in vivo* therapeutic model. Administration of the antitumor agents was initiated when the tumor volume reached ~500 mm^3^ (day 0), and continued on days 2 and 4, and the mice were sacrificed on day 7.

C3H/HeJ mice were employed for the therapeutic model as they are H2-matched to LM8 since this cell line originated from that strain of mouse (14). Mice were obtained from CLEA Japan Inc. (Tokyo, Japan) and bred at the Department of Biological Resources, Ehime University Integrated Center of Science. LM8 cells (3.0×10^6^ cells per mice) were subcutaneously transplanted into 6-week-old male C3H/HeJ mice. After ~3 weeks, when the tumor volume reached ~500 mm^3^, administration of the therapeutic agents was initiated. A scheme of the administration protocol is presented in Fig. 1.

Combinations of the agents were administered as follows: PBS (i.v., sham administration), CV (0.1 mg/g BW), IFO (direct i.v. 0.1 mg/g BW), IV (i.v. 0.1 mg/g BW), IV+CSB, and IV+CV. Five to eight animals were tested in each group. Agents were given by tail vein injection (~50 μl) on days 0, 2 and 4, and then the mice were sacrificed on day 7 under anesthesia. Tumor diameter and body weight were evaluated every day. After the harvest, tumor volumes and tumor weights were evaluated, and the entire organs were processed for histopathological analyses. All the animal experimental protocols were in accordance with the Guide for Animal Experimentation, Ehime University, and approved by the Committee for Animal Experimentation, Ehime University.

### Histopathological analyses

Tumors and entire organs from the harvested mice were formalin-fixed and paraffin-embedded. The viability of the tumor tissue was evaluated as the area of viable tumor. In addition, entire organ tissue sections stained with hematoxylin and eosin, periodic acid-Schiff (PAS), and Elastica-Goldner were screened by a skilled pathologist to determine the adverse effects.

### Fertility test

A fertility test was performed as follows. Three male C3H/HeJ mice in each group that were administered IFO, IV or IV+CV were cross-mated with 6-week-old female C3H/HeJ mice, and the fertility of each animal was evaluated.

### Statistical analyses

Body weight, tumor volume, and viability of the tumor at the maximum sections were statistically compared among the different groups with the Student’s t-test, using GraphPad Prism^®^ software (GraphPad Software Inc., La Jolla, CA, USA).

## Results

### Antitumor effects in vitro

**Figure 2:**
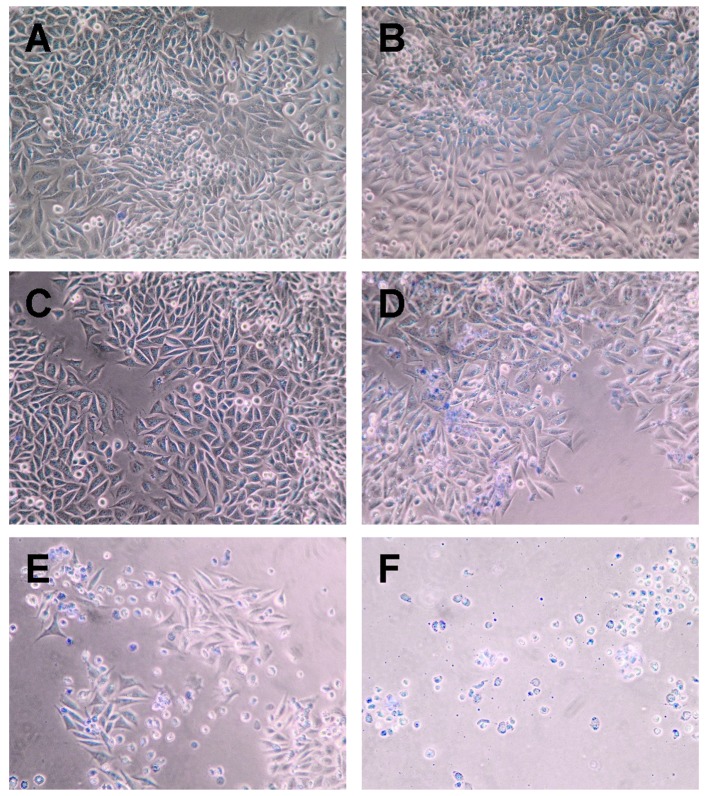
Representative photomicrographs of the trypan blue-stained LM8 cells after a 2-h incubation with antitumor agents: (A) PV, (B) CV, (C) IFO, (D) IV, (E) IFO+CV and (F) IV+CV.

*In vitro* analyses showed that cultured LM8 mouse osteosarcoma cells treated with IV+CV manifested almost complete cell death by the trypan blue stain assay, while PBS, CSB, PV and CV showed almost no cell death. IFO resulted in 13%, IV resulted in 28%, and IFO+CV resulted in 75% cell death (Fig. 2 and Table I).

### Apoptosis analyses in vitro

**Figure 3:**
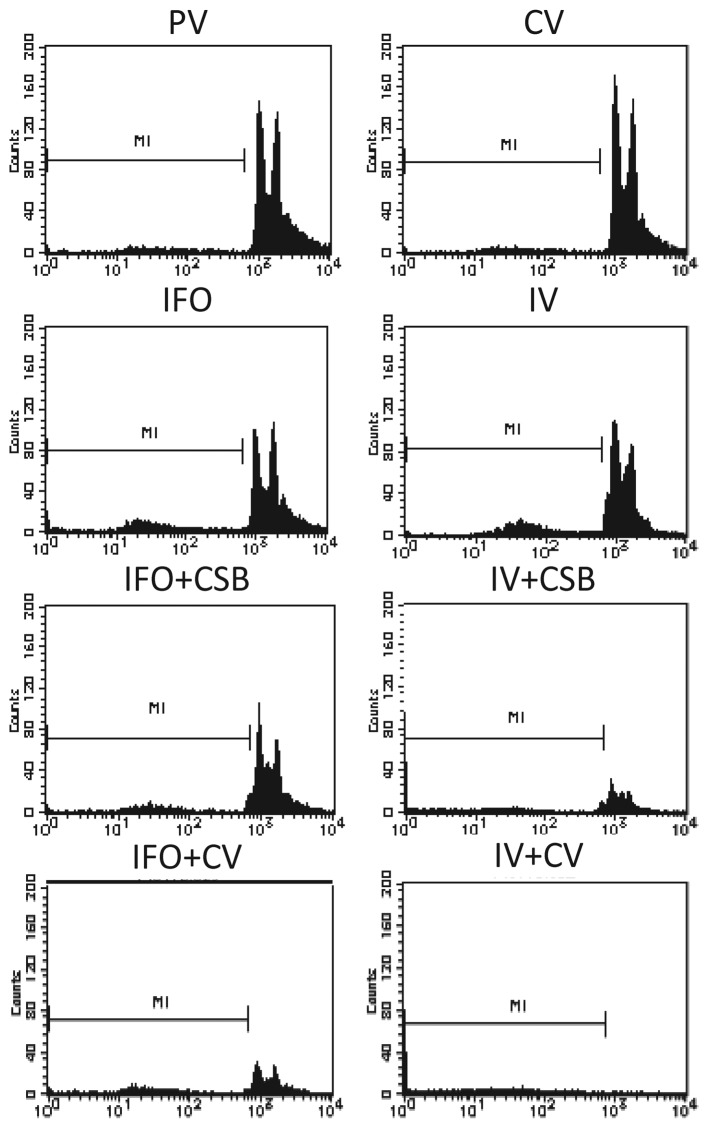
Flow cytometric analyses of apoptosis using PI staining. Each graph shows the event count at each intensity according to the antitumor agent. The proportion of the population (%) of apoptotic and/or necrotic cells was measured as M1 and is presented in Table II.

By PI analysis, almost all the cells treated with IV+CV underwent cell death by apoptosis. In contrast, PBS, CSB, PN and CV induced very limited cell death, whereas IFO and IV induced apoptosis or necrosis in a small population (8.8–10.2%), and IFO+CSB and IV+CSB increased the cell death effect to approximately a quarter to one-third of the population (Fig. 3, Table II).

### Antitumor effects in vivo

**Figure 4:**
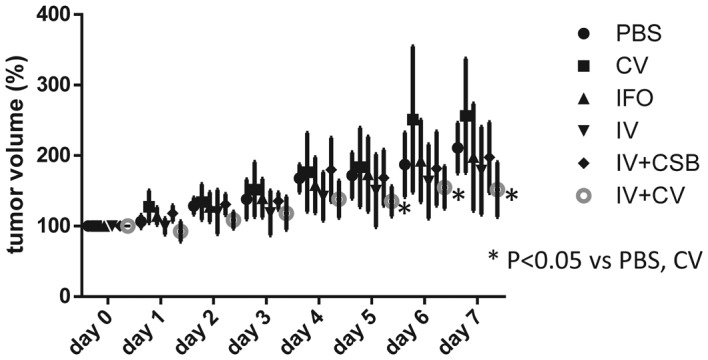
Trace of tumor volumes after antitumor agent administration. The symbols represent the mean value of each group, and the bars represent standard deviation.

The body weights were not significantly different among the different groups. The tumor volumes in the IV+CV group were significantly reduced as compared to those of the control groups (PBS and CV), and showed a tendency towards a decrease compared with the PV and IFO direct i.v. groups on days 5–7 (Fig. 4).

**Figure 5:**
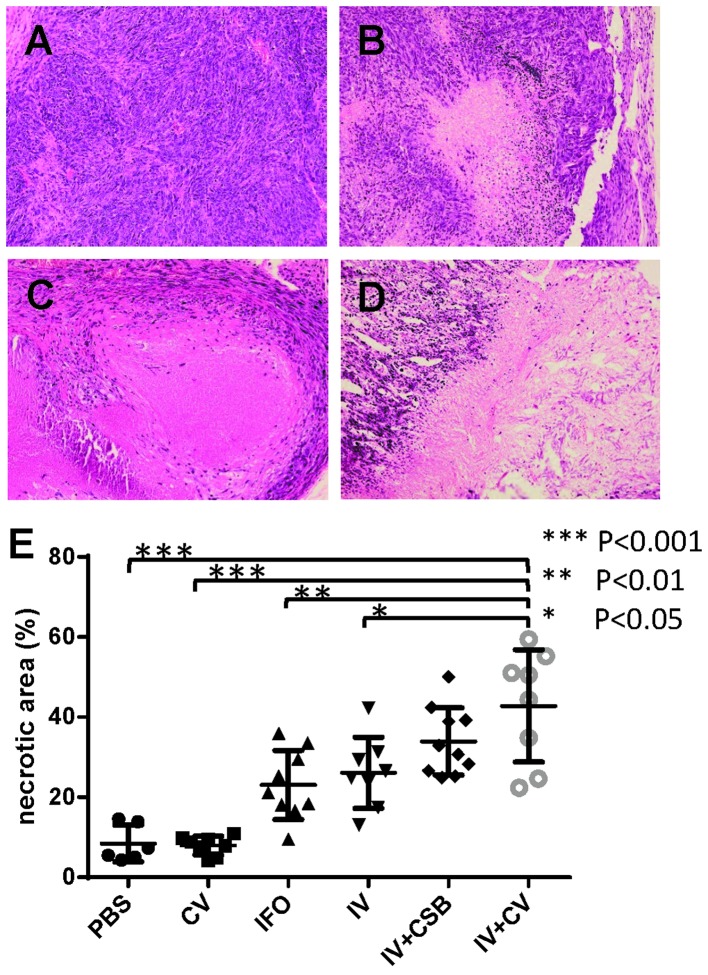
Representative photomicrographs of the tumors treated with (A) CV, (B) IFO, (C) IV and (D) IV+CV, and the proportion of necrotic area (%) of each animal from each group. Center bars represent mean value, and the upper and lower bars represent standard deviation.

The histopathological analyses of the tumors demonstrated that IFO, IV, IV+CSB, and IV+CV groups showed significantly larger non-viable tumor areas as compared to the controls. In addition, the IV+CV group manifested significantly greater necrotic areas compared with the IFO and IV groups (Fig. 5).

### Histopathological analyses for adverse effects in vivo

**Figure 6:**
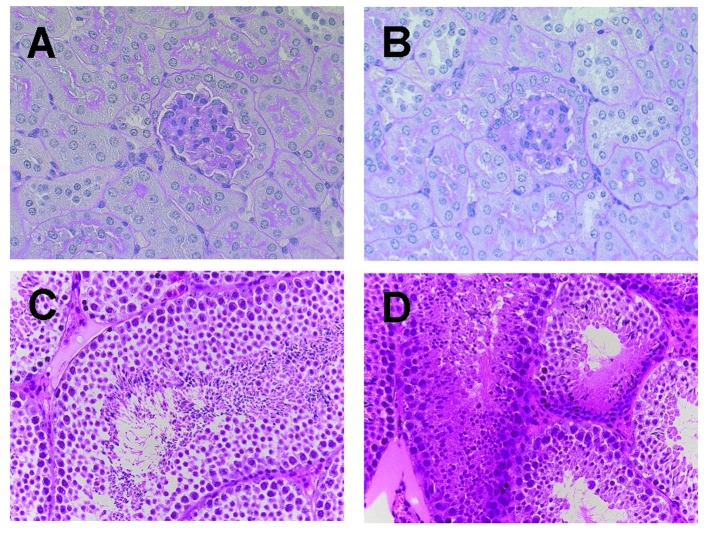
Representative photomicrographs of the kidney (A and B) and testis (C and D) from an animal treated with IV+CV (A and C) and IFO i.v. (B and D).

To clarify whether the Span 80 vesicles can prevent adverse effects induced by chemotherapeutic agents, histopathological examination of the entire organs was performed. Marked differences were noted in the kidney and testis. In the kidney, marked tubular injury, which was manifested as loss of brush border, as well as glomerular changes such as expansion of the mesangial matrix were observed in the IFO direct i.v. group as compared to those in the IV and IV+CV groups (Fig. 6A and B). In addition, the IFO direct i.v. group showed marked suppression of spermatogenesis with necrosis of the germ cells, while the IV and IV+CV groups showed no marked changes in spermatogenesis (Fig. 6C and D).

### Fertility test

To clarify whether the DDS using Span 80 nano-vesicles could prevent infertility, a fertility test was performed. The test revealed that the male mice after IV+CV administration had normal fertility, and there were no malformations in their progeny (data not shown).

## Discussion

We present findings herein to support the evidence that the DDS with Span 80 vesicles can enhance the antitumor effects of IFO and caffeine-potentiated IFO chemotherapy, as well as suppress the adverse effects induced by chemotherapy. *In vitro*, IV+CV induced drastic cell death in a very early phase in contrast to the ‘mild’ apoptosis induction by IFO alone, IV alone or combinations of IFO+CSB, IV+CSB and IFO+CV. This suggests that the immediate delivery of IFO and CSB into the cytosol leads to extremely rapid apoptosis induction, although the detailed mechanisms are still unknown.

The DDS with nano-vesicles has been developed with many types of phospholipids and/or detergents (6–8,15). Among them, the Span 80 nano-vesicle appears to be a promising material due to its favorable physicochemical properties, such as membrane fluidity and flexibility. Regarding membrane fluidity, Span 80 vesicles were reported to have markedly high fluidity with various cholesterol contents as compared to conventional phospholipid liposomes, including 1,2-dipalmitoyl-sn-glycero-3-phosphocholine (DPPC) and 1-palmitoyl-2-oleoyl-sn-glycero-3-phosphocholine (POPC) liposomes. In addition to the high fluidity, Span 80 vesicles also have greater flexibility as compared to DPPC and POPC liposomes (10).

Kato *et al* reported that while non-vesicular aggregates are often observed in liposome suspensions, Span 80 vesicle suspensions can also include non-vesicular aggregates such as tubulin structures. Meanwhile, it has been reported that Span 80 vesicles are kinetically-trapped aggregates and not thermodynamic equilibrium structures, as in most vesicles formed from phosphatidylcholines (liposomes) (9).

IFO has been reported to induce adverse effects in the kidney (16–20), gonadal cells (21–23), and bone marrow along with other organs (24,25). In the present study, the mice in the IFO i.v. group also exhibited a certain degree of tubular injury and glomerular changes as well as severe suppression of spermatogenesis with gonadal cell necrosis. In contrast, DDS with Span 80 vesicles manifested marked suppression of the adverse effects in the kidney and testis. This phenomenon could be due to tumor selectivity of the vesicles, which may be partially derived from the avoidance of phagocytosis by macrophages based on the pegylation of the vesicles, and could also be from the lower permeability of vesicles at the blood-testis barrier as compared to the direct injection of low molecular weight molecules such as IFO (23,26).

Our findings suggest that the higher tumor selectivity of Span 80 vesicles may be associated with their pegylation as well as high fluidity and flexibility, which result in higher vascular permeability and tendency to fuse with the instable cell membrane of the tumors (27). Indeed, a cell fusion model of Span 80 vesicles has been recently reported (11). In addition, our data demonstrated that CV produced significantly greater antitumor effects as compared to the direct injection of CSB. This may also be due to pegylation-associated tumor selectivity and the tendency for cell fusion which may enable the immediate delivery of caffeine into the cytoplasm. Regarding the selectivity of caffeine delivery, it may result in the prevention of caffeine toxicity which may cause the withdrawal of numerous patients from caffeine-potentiated chemotherapy (5).

Currently, DDS using liposomal nano-vesicles containing doxorubicin is applied with marked efficacy (6–8,28–30). Next generation liposomes, which have targeting molecules on their surface, have also been under development. A study of the anti-carcinoma application of Span 80 vesicles containing doxorubicin was recently reported (27). Span 80 not only has favorable physicochemical properties, but is also safe since it is already used as a stabilizer for injected drugs. In addition, Span 80 vesicles should cost drastically less than normal liposomes. Hence, Span 80 vesicles could be a promising next generation material for DDS.

In summary, the DDS with Span 80 vesicles may enhance the antitumor effects of IFO and of caffeine-potentiated IFO chemotherapy against osteosarcoma. Moreover, the usage of this DDS may suppress the adverse effects of chemotherapy. Thus, this DDS model has potential importance for clinical application in the therapy of metastatic osteosarcoma.

## Figures and Tables

**Table I:** Non-viable cell population in trypan blue analysis.

Treatment	Population of non-viable cells (%)(mean ± SD)
PBS	1.5 ±0.9
CSB	2.1±1.2
PV	3.3±1.8
CV	3.1±1.9
IFO	13±3.4^a^
IV	28±5.5^a^
IFO+CSB	25±6.7^b^
IV+CSB	40±9.2^c^
IFO+CV	75±10.8^d^
IV+CV	98±1.2^e^

a P<0.05 vs. PBS, CBS, PV and CV;

b P<0.05 vs. PBS, CBS, PV, CV and IFO;

c P<0.01 vs. IFO groups, P<0.05 vs. IV and IFO+CBS;

d P<0.001 vs. IV+CBS, P<0.05 vs. IFO+CV.

**Table II:** Population of non-viable cells (M1) in flow cytometric analysis.

Treatment	Population of non-viable cells (%)
PV	1.6±1.1
CV	1.4±1.0
IFO	8.8±1.9^a^
IV	10.2±2.9^a^
IFO+CSB	16.5±3.9^b^
IV+CSB	25.2±4.2^b^
IFO+CV	32.8±5.9^b^
IV+CV	97.1±1.9^c^

a P<0.05 vs. PV and CV;

b P<0.05 vs. PV and CV;

c P<0.001 vs. PV and CV.
